# HSF1 Alleviates Brain Injury by Inhibiting NLRP3-Induced Pyroptosis in a Sepsis Model

**DOI:** 10.1155/2023/2252255

**Published:** 2023-01-27

**Authors:** Yi-fu He, Xi-min Hu, Md. Asaduzzaman Khan, Bo-yao Yu, Yi-cun Sheng, Xian-zhong Xiao, Xin-xing Wan, Si-pin Tan, Kun Xiong

**Affiliations:** ^1^Department of Obstetrics and Gynecology, Xiangya Hospital, Central South University, Changsha 410008, China; ^2^Clinical Medicine Eight-Year Program, Xiangya School of Medicine, Central South University, Changsha 410013, China; ^3^The Research Centre for Preclinical Medicine, Southwest Medical University, Luzhou 646000, China; ^4^Clinical Medicine Five-Year Program, Xiangya School of Medicine, Central South University, Changsha 410013, China; ^5^Key Laboratory of Sepsis Translational Medicine of Hunan, Department of Pathophysiology, Xiangya School of Medicine, Central South University, Changsha 410013, China; ^6^Department of Endocrinology, Third Xiangya Hospital, Central South University, Changsha 410013, China; ^7^Department of Anatomy and Neurobiology, School of Basic Medical Science, Central South University, Changsha 410013, China; ^8^Hunan Key Laboratory of Ophthalmology, Changsha 410008, China; ^9^Key Laboratory of Emergency and Trauma, Ministry of Education, College of Emergency and Trauma, Hainan Medical University, Haikou 571199, China

## Abstract

**Background:**

Sepsis, which could cause a systemic inflammatory response, is a life-threatening disease with a high morbidity and mortality rate. There is evidence that brain injury may be related to severe systemic infection induced by sepsis. The brain injury caused by sepsis could increase the risk of mortality in septic patients, which seriously affects the septic patient's prognosis of survival. Although there remains a focus on sepsis research, clinical measures to prevent and treat brain injury in sepsis are not yet available, and the high mortality rate is still a big health burden. Therefore, it is necessary to investigate the new molecules or regulated pathways that can effectively inhibit the progress of sepsis.

**Objective:**

NLR family pyrin domain-containing 3 (NLRP3) increased in the procession of sepsis and functioned as the key regulator of pyroptosis. Heat shock factor 1 (HSF1) can protect organs from multiorgan dysfunction syndrome induced by lipopolysaccharides in mice, and NLRP3 could be inhibited by HSF1 in many organs. However, whether HSF1 regulated NLRP3 in sepsis-induced brain injury, as well as the detailed mechanism of HSF1 in brain injury, remains unknown in the sepsis model. In this research, we try to explore the relationship between HSF1 and NLRP3 in a sepsis model and try to reveal the mechanism of HSF1 inhibiting the process of brain injury.

**Methods:**

In this study, we used wild-type mice and *hsf1*^−/−^ mice for *in vivo* research and PC12 cells for *in vitro* research. Real-time PCR and Western blot were used to analyze the expression of HSF1, NLRP3, cytokines, and pyrolytic proteins. EthD-III staining was chosen to detect the pyroptosis of the hippocampus and PC12 cells.

**Results:**

The results showed that HSF1 is negatively related to pyroptosis. The pyroptosis in cells of brain tissue was significantly increased in the *hsf1*^−/−^ mouse model compared to *hsf1*^+/+^ mice. In PC12 cells, *hsf1* siRNA can upregulate pyroptosis while HSF1-transfected plasmid could inhibit the pyroptosis. HSF1 could negatively regulate the NLRP3 pathway in PC12 cells, while *hsf1* siRNA enhanced the pyroptosis in PC12 cells, which could be reversed by *nlrp3* siRNA.

**Conclusion:**

These results imply that HSF1 could alleviate sepsis-induced brain injury by inhibiting pyroptosis through the NLRP3-dependent pathway in brain tissue and PC12 cells, suggesting HSF1 as a potential molecular target for treating brain injury in sepsis clinical studies.

## 1. Introduction

Sepsis is a life-threatening disease with a high morbidity and mortality rate [1, 2]. The pathogenesis of sepsis is extremely complicated due to the dysregulation of the body's response to infection [3–5]. In sepsis, immune dysfunction, coagulation dysfunction, and systemic inflammatory network effect induce damage to its tissues and organs [6–9]. The mechanism of brain impairment among patients with sepsis was still unclear, and brain injury could increase the risk of mortality in septic patients. There was evidence that brain injury may be related to severe systemic infection induced by sepsis, which could cause a systemic inflammatory response [10–13]. However, the exact pathophysiology of brain injury in sepsis was complex, and the possible processes cause brain injury by increasing the expression of proinflammatory cytokines (interleukin (IL) family), including oxidative stresses and the damaging of the blood-brain barrier (BBB) structure [14–16]. Therefore, it is necessary to investigate the new molecules or regulated pathways that can effectively inhibit the progress of sepsis to form brain injury.

In recent years, heat shock proteins (HSPs) have become an interesting topic in the development of new treatments for sepsis [17]. The induction of HSPs responsible for maturation, antioxidative protection, adiposis, etc., can be significantly affected by harmful stress such as reactive oxygen species (ROS) and inflammation [18–20]. Interacting with other signaling pathways, HSPs can produce a collective response against harmful stress like sepsis [21–25]. HSPs are evolutionarily conserved and involved in the cellular protective mechanism of heat shock response (HSR) to maintain protein homeostasis in almost all eukaryotic cells. Heat shock factor 1 (HSF1) plays a key role in the HSR, and thus, HSP expression is highly dependent on HSF1 regulation [26–28]. HSF1 can spontaneously interact with a complex of chaperone proteins HSP90, HSP70, and HSP40, preventing it from binding to DNA. Facts have proved that HSF1 can protect organs from multiorgan dysfunction syndrome induced by lipopolysaccharides (LPS) [29]. Furthermore, inhibiting HSF1 in mice prevents HSP induction and makes cells vulnerable to proteotoxic stress [23]. It is evident that HSF1 decreases the production of inflammatory mediators to attenuate the inflammatory responses caused by LPS, and HSF1 exerts protective effects against brain dysfunction in sepsis [30]. However, the exact mechanism needs to be clarified.

The NLRP3 inflammasome mainly exists in immune and inflammatory cells following inflammatory activation [31–33]. NLRP3 can respond to various stimuli such as viral RNA, lysosomal damage, and ROS [34–36]. The NLRP3 inflammasome increased in the procession of sepsis and functioned as the key regulator of pyroptosis [37–39]. Pyroptosis, playing a protective role in host defense during infection, is an inflammatory form of regulated cell death (RCD) [40–45]. Mature IL-1*β* is cleaved by the activated NLRP3/caspase1 pathway, and the cleaved IL-1*β* is released through the pore in the cell membrane and induces pyroptosis [46–48]. Abnormal pyroptosis is harmful to normal cells and organs, and so it is necessary to keep the pyroptosis within the limits of homeostasis. Downregulated NLRP3 can suppress pyroptosis and protect the endothelium from early sepsis, and the inhibition of the NLRP3 inflammasome can prevent sepsis [38, 49]. Moreover, HSF1 can regulate the innate immunity of the NLRP3 inflammasome, leading to the protection of sepsis [50]. HSF1 could protect many organs such as the liver, lung, and kidney in the sepsis animal model. However, whether HSF1 could alleviate brain injury in sepsis remains revealed [51]. The clinical measures to prevent and treat brain injury induced by sepsis are not yet available, which is still a big health burden in treating sepsis [12–15]. It is very important to find a possible molecular pathway for alleviating pyroptosis through NLRP3 in brain tissue and nerve cell lines, which can shed light on the mechanism to protect brain tissue in sepsis.

## 2. Materials and Methods

### 2.1. Cell Culture

PC12 pheochromocytoma cells were cultured in DMEM culture media (Life Technologies, Carlsbad, CA, USA) supplemented with 10% fetal bovine serum (FBS; Life Technologies) at 37°C, 5% CO_2_. When the PC12 cells had grown to approximately 80% density, they were subjected to LPS-different times (a 12 h point was chosen for further research)/adenosine triphosphate (ATP)-1 h treatment, including small interfering RNA (siRNA) or plasmid transfection, respectively.

### 2.2. Cell Transfection

Cells (*n* = 5 for each group) were transfected with mouse HSF1-overexpressing plasmids (GeneChem, Shanghai, China) and siRNA as described previously [52]. When the PC12 was approximately 70% to 80% confluent, the siRNA and plasmids were transfected with Lipofectamine 3000 (Invitrogen, Carlsbad, CA, USA).

### 2.3. Real-Time Reverse Transcription-Polymerase Chain Reaction (PCR)

The total RNA of the PC12 (*n* = 5 for each group) or hippocampus (*n* = 3 for each group) was isolated with the TRIzol reagent (Life Technologies), and reverse transcription was performed using oligo-dT primers with Superior III RT Supermix (Innogene Biotech, Beijing, China). Real-time PCR was performed using the Eppendorf realplex with UltraSYBR Mixture (Toyobo Co., Osaka, Japan), and the expression was quantified compared to the housekeeping gene (*β*-actin) for mRNA. The primer sequences used for qPCR were as follows (Table 1).

### 2.4. Western Blot Assay

The total protein of the PC12 (*n* = 5 for each group) or hippocampus (*n* = 3 for each group) was extracted and separated on a 12% sodium dodecyl sulfate-polyacrylamide gel electrophoresis (SDS-PAGE) with a constant voltage of 75 V. The electrophoresed proteins were transferred to nitrocellulose membranes with a transfer apparatus (Bio-Rad, Hercules, CA, USA). The membranes were blocked with 5% nonfat milk in Tris-buffered saline (pH 7.5) with 0.05% Tween 20 for 2 hours at room temperature. Primary antibodies, including HSF1, NLRP3, IL-1, caspase1, and GAPDH (Proteintech, Chicago, IL, USA), were diluted to 1 : 1000 in TBS buffer overnight at 4°C. The blots were washed 3 times in TBS buffer for 10 min and then immersed in the secondary antibody solution containing the goat-IgG rabbit polyclonal antibody (1 : 5000 in TBS buffer, Proteintech) for 2 h and diluted with TBS buffer. The membranes were washed 3 times for 10 min in TBS buffer. The immunoblotted proteins were visualized using an ECL Western blot luminol reagent (Advansta, Menlo Park, CA, USA) and quantified using a Universal Hood II chemiluminescence detection system (Bio-Rad) by imaging scans.

### 2.5. Brain Tissue of Animals

The brain tissues of *hsf1* KO (*hsf1*^−/−^) and wild-type (*hsf1*^+/+^) mice were provided by Prof. Xiao Xian-zhong (The Department of Pathophysiology, School of Basic Medical Science, Central South University, Changsha, Hunan, China) and have been described previously [30, 52]. Protocols for animal breeding and experiments were approved by the Institutional Animal Care and Use Committee of Central South University under license number 2018sydw0378 (approval date: 25 Nov. 2018). 16-20-week-old (weight 20-25 g) mice were subjected to CLP or sham.

### 2.6. Cecal Ligation and Puncture Model

Cecal ligation and puncture (CLP) was performed as previously described in Xiao's lab [30, 52]. Briefly, *hsf1*^−/−^ and *hsf1*^+/+^ mice (*n* = 3 for each group) were anesthetized with 2% isoflurane. Under aseptic conditions, a midline laparotomy incision was performed to allow exposure of the cecum. One-third of the cecum was tightly ligated with an orientation distal to the ileocecal valve and was perforated twice with a 22-gauge needle. A few feces were extruded from the puncture holes to ensure patency. The laparotomy was then sutured, followed by fluid resuscitation (normal saline, 50 mL/kg). The mice were euthanized after 12 h, and the hippocampus was removed and fixed with paraformaldehyde or stored at -80°C for further research.

### 2.7. Immunofluorescence Staining

The brain tissues were collected and fixed in 4% paraformaldehyde and embedded in paraffin for preparation of 4 *μ*m thick tissue sections. The dewaxed paraffin sections of hippocampal tissue from differently treated mice were detergent extracted with 0.1% Triton X-100 for 10 min at 4°C before being incubated overnight with the HSF1 (sc-13516, Santa Cruz, Dallas, Texas, USA) and NLRP3 (19771-1-AP, Proteintech) polyclonal antibody (1 : 100) at 4°C. The slide was washed with phosphate-buffered saline (PBS) and incubated with 150 *μ*L with the FITC (SA00003-11)- or Cy3 (SA00009-2)-tagged secondary antibody (1 : 200) at room temperature (RT) for 2 hours. The slide was then incubated with an immunofluorescence polyclonal antibody for 1 h. The nuclei were stained with DAPI (Sigma-Aldrich, St. Louis, MO, USA). The laser scanning microscope in Servicebio, Wuhan, China, was used to capture the image. The proportion of red-positive/green-positive cells to blue-positive cells was calculated using ImageJ (NIH, Baltimore, MD, USA).

### 2.8. EthD-III Staining

Pyroptosis was assessed by staining with EthD-III (Biotium, Fremont, CA, USA) as previously described [53, 54]. In brief, PC12 cells were treated with the corresponding stimuli or unfixed brain tissue sections from differently treated mice that were incubated with EthD-III (10 *μ*g/mL) at room temperature (RT) for 10 min and washed with PBS for 3 × 5 min. Afterward, they were fixed with 4% paraformaldehyde (PF) for 20 min and washed three times with ice-cold PBS. Hoechst 33258 (APExBIO, Boston, MA, USA) was used for nuclear staining at RT for 10 min. Afterward, the samples were washed with PBS for 3 × 5 min before the coverslips were finally sealed by a drop of glycerin (Solarbio, Beijing, China) and visualized, choosing the hippocampus or 3~6 positions in PC12 cells under a fluorescence microscope (Olympus, Tokyo, Japan). The laser scanning microscope was used to capture images. The proportion of red-positive cells to blue-positive cells was calculated using ImageJ.

### 2.9. Statistical Analysis

SPSS19.0 was used for statistical analysis. The results were summarized and presented as mean ± standard deviation (SD) and analyzed using an independent *t*-test for the two groups. One-way analysis of variance (one-way ANOVA) was utilized for comparing multiple groups, followed by multiple comparison tests (Bonferroni *post hoc* tests). The statistically significant level was defined as *p* < 0.05.

## 3. Results

### 3.1. Increased Expression of HSF1 in Hippocampal Tissue of Septic Mice

**Figure 1:**
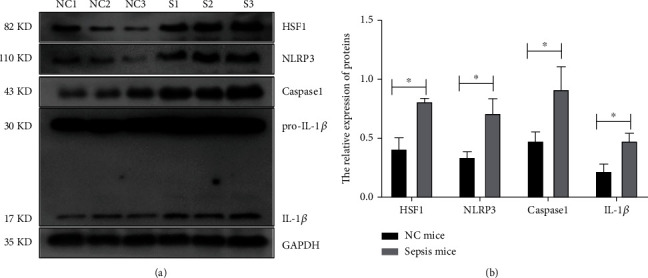
Expression of HSF1, cytokines, and pyrolytic proteins in the hippocampus. (a) Western blot analysis was performed to detect the expression of the inflammatory cytokines and pyrolytic proteins in hippocampal tissue. NC1-NC3: negative control group (sham group); S1-S3: sepsis group. (b) WB images were quantified, and their statistical analysis is shown in the graph. Experiments were repeated independently, ^∗^*p* < 0.05.

The previous results [52] had proved the increased expression of HSF1 in the lung, kidney, and liver of septic mice constructed by CLP, but the expression of HSF1 in the hippocampus of CLP septic mice remained unknown. In this research, we collected the hippocampal tissue of HSF1 wild-type (*hsf1*^+/+^) control mice and septic mice constructed by CLP. Expression of HSF1 in hippocampal tissue was detected by Western blot. As the results showed, HSF1 obviously increased in the CLP sepsis model (Figure 1). Because the exact mechanism of HSF1 in the process of sepsis still had not been demonstrated, the expression of inflammatory factors was further assessed in the hippocampal tissue of *hsf1*^+/+^ mice. The results revealed that the expression of inflammatory cytokines such as caspase1 and NLRP3 was significantly elevated as well as HSF1, and the cleavage of IL-1*β* was also increased in CLP septic mice (Figure 1).

### 3.2. Pyroptosis Is Elevated in Brain Tissue of *hsf1*^−/−^ Septic Mice

**Figure 2:**
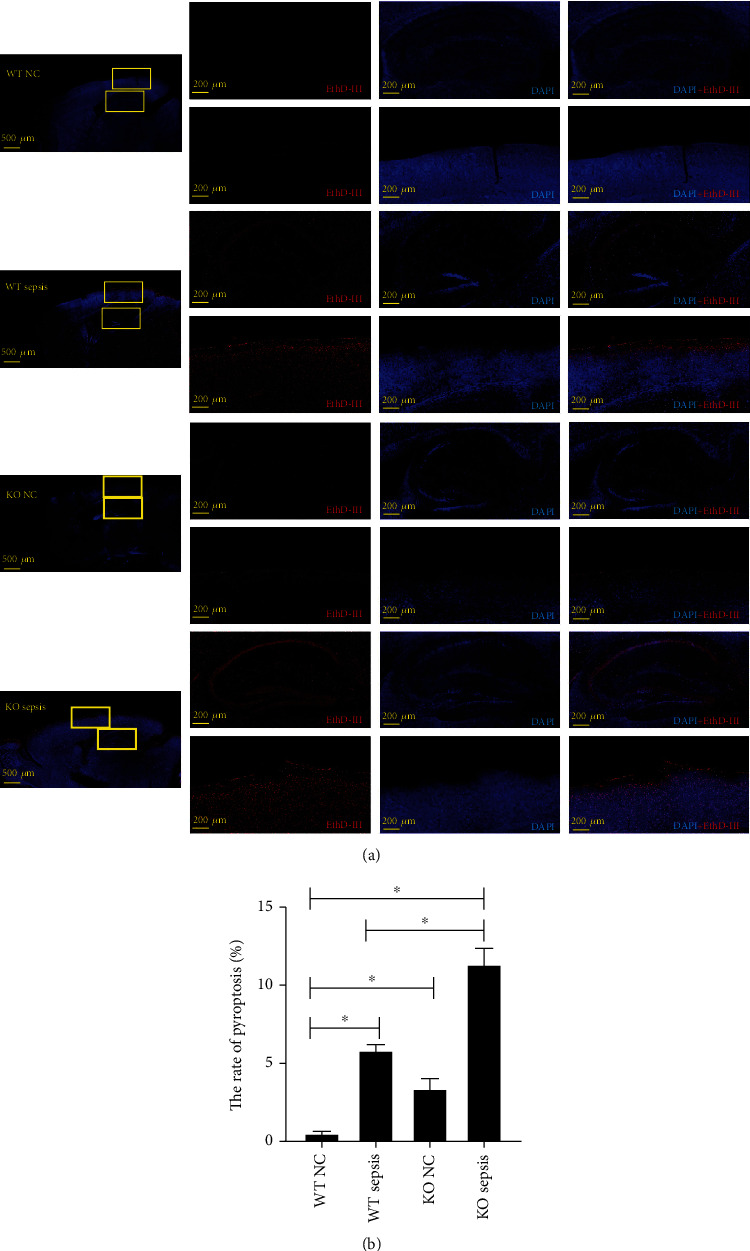
Pyroptosis in brain tissues of different mouse groups. (a) EthD-III staining (red) was labeled as pyroptosis, and DAPI staining (blue) was tagged as nuclei. The photos were taken by the laser scanning microscope at 500 *μ*m (apply to the first column from the left side) and 200 *μ*m (apply to the second to fourth columns from the left side). (b) The statistical analysis of pyroptosis is shown in the graph. Experiments were repeated independently, ^∗^*p* < 0.05. WT NC: wild-type negative control group; WT S: wild-type sepsis group; KO NC: *hsf1*^−/−^ negative control group; KO S: *hsf*1^−/−^ sepsis group.

The inflammatory molecules such as IL-1*β*, caspase1, and NLRP3 played important roles in pyroptosis [46, 55]. As the expression of HSF1 in the CLP sepsis model was related to IL-1*β*, caspase1, and NLRP3 (Figure 1), pyroptosis in the brain tissue of differently treated mice was detected. The EthD-III staining experiments found that EthD-III-positive cells existed in the dentate gyrus and pyramidal cells in hippocampal and cortex tissues. The pyroptotic cells looked like cabbage or fried egg, with the nucleus located in the center [25]. The results showed that the level of pyroptosis was elevated in both the hippocampal and cortex tissues of septic *hsf1*^−/−^ and *hsf1*^+/+^ CLP models contrasted to the sham control, respectively. Interestingly, the pyroptosis in hippocampal and cortex tissues was found to increase in the *hsf1*^−/−^ CLP model compared to the *hsf1*^+/+^ CLP model, while the *hsf1*^−/−^ mice also showed more pyroptosis in hippocampal and cortex tissues than *hsf1*^+/+^ mice (Figure 2).

### 3.3. *hsf1*^−/−^ Septic Mice Displayed Enhanced NLRP3 Expression in the Brain

**Figure 3:**
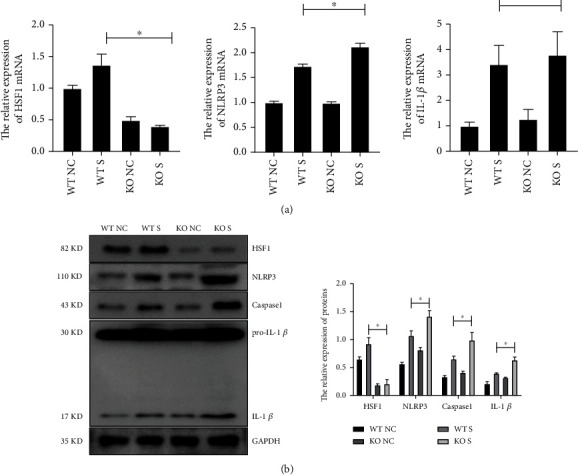
Expression of NLRP3 in the hippocampus. (a) Real-time PCR was used to detect the expression of the inflammatory cytokines and pyrolytic proteins in hippocampal tissue. Experiments were repeated independently, ^∗^*p* < 0.05. (b) Western blot was used to detect the expression of the inflammatory cytokines and pyrolytic proteins in hippocampal tissue. WT C: wild-type control group; WT S: wild-type sepsis group; KO C: *hsf1*^−/−^ control group; KO S: *hsf1*^−/−^ sepsis group. The statistical analysis of WB is shown in the graph. Experiments were repeated independently, ^∗^*p* < 0.05.

**Figure 4:**
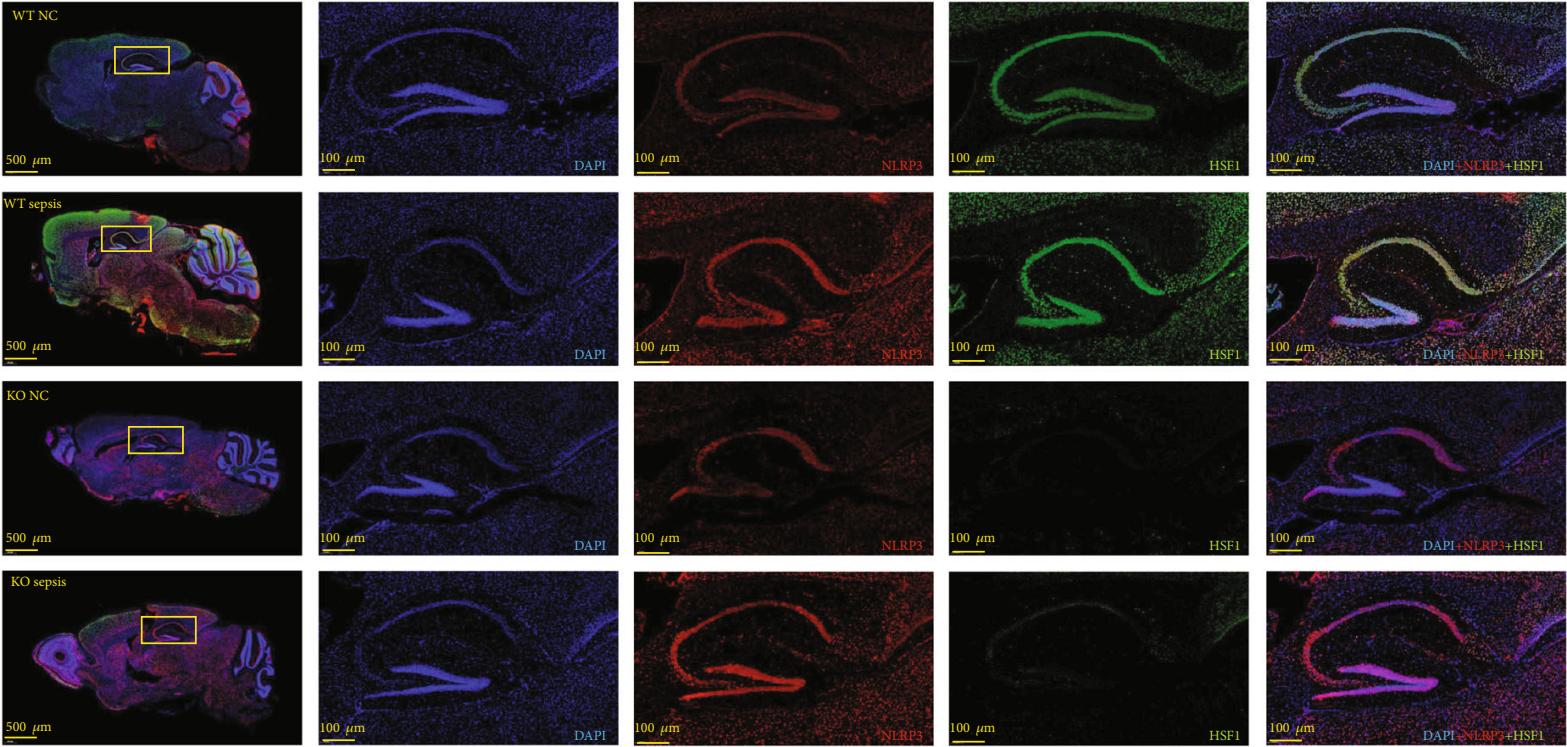
Immunofluorescence staining of NLRP3. Blue light: DAPI; FITC green light: HSF1; Cy3 red light: NLRP3. The photos were taken by the laser scanning microscope at 500 *μ*m (apply to the first column from the left side) and 100 *μ*m (apply to the second to fifth columns from the left side).

The CLP sepsis model and sham model were established in *hsf1*^+/+^ mice and *hsf1*^−/−^ mice, and the models were sentenced to death after 12 h. Brain tissue was collected and divided into two parts. One hemisphere containing hippocampal tissue was used in Western blot and real-time PCR, while another hemisphere was used in immunofluorescence staining.  Figure 3 reveals that the mRNA and protein level of NLRP3 was elevated in the hippocampus of both *hsf1*^−/−^ and *hsf1*^+/+^ CLP mice, and the expression of NLRP3 protein was increased in the *hsf1*^−/−^ CLP mice compared to *hsf1*^+/+^ CLP mice. The immunofluorescence experiments were also proved to significantly increase NLRP3 in the brain tissue of *hsf1*^−/−^ septic mice contrasted to *hsf1*^+/+^ septic mice (Figure 4). HSF1 could protect organs from multiorgan dysfunction syndrome induced by LPS [56]. Combined with the decreased level of pyroptosis in *hsf1*^+/+^ CLP mice compared to *hsf1*^−/−^ CLP mice (Figure 2), we supposed that HSF1 might alleviate brain injury by inhibiting NLRP3-dependent pyroptosis in the brain of the sepsis model.

### 3.4. HSF1 Negatively Regulated NLRP3 and Pyroptosis in PC12 Cells *In Vitro*

**Figure 5:**
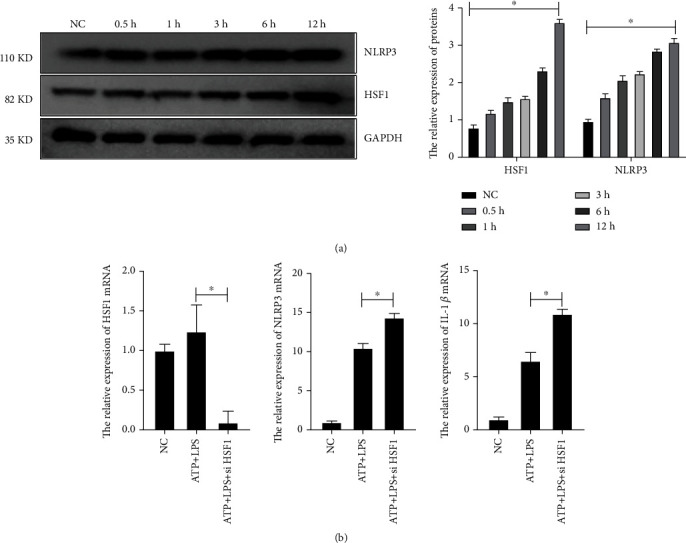
HSF1 and NLRP3 expression in LPS+ATP-treated PC12 cells. (a) Western blot was used to detect the expression of the NLRP3 and HSF1 in PC12 cells at different times treated by LPS+ATP. The statistical analysis of WB is shown in the graph. Experiments were repeated independently, ^∗^*p* < 0.05 vs. negative control. (b) qPCR detected the expression of HSF1, NLRP3, and IL-1*β* in PC12 cells treated with LPS+ATP. Experiments were repeated independently, ^∗^*p* < 0.05 vs. negative control.

**Figure 6:**
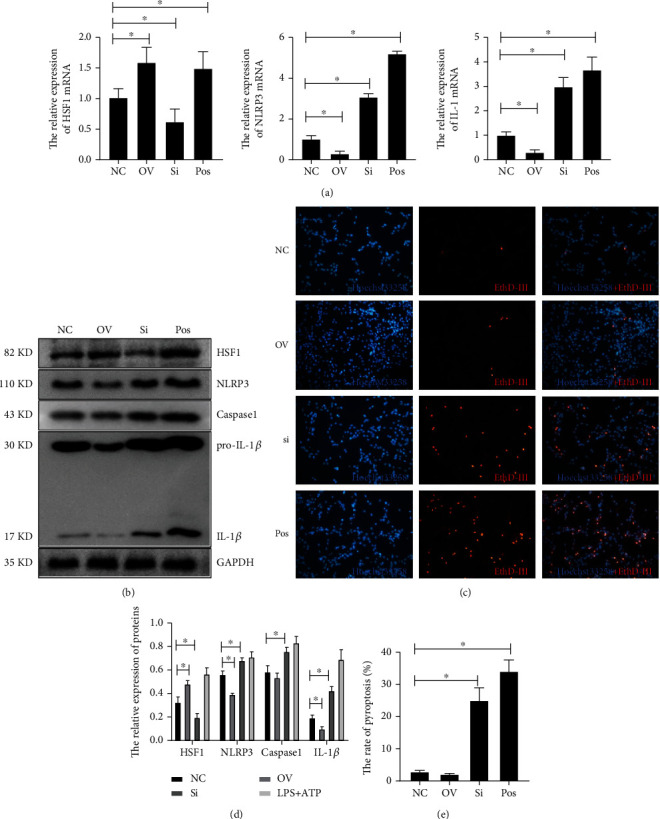
HSF1 regulates the pyroptosis of PC12 and the expression of NLRP3. (a) Real-time PCR was used to detect the expression of HSF1, NLRP3, and IL-1*β* in PC12 cells. (b, d) Western blot was used to detect the expression of the inflammatory cytokines and pyrolytic proteins in differently treated PC12, ^∗^*p* < 0.05. (c, e) EthD-III staining (red) was labeled as pyroptosis, and Hoechst staining (blue) was tagged as nuclei, ^∗^*p* < 0.05. NC: negative control group; OV: *hsf1* overexpression group; si: *hsf1* silence group; LPS+ATP: PC12 cells treated with LPS for 12 hours and ATP for 1 h. Scale bar: 100 *μ*m, apply to all the panels.

To reveal the alleviating mechanism of HSF1 on sepsis in CNS, the PC12 cell model was used for cell experiments. LPS is usually used to induce inflammation. The LPS+ATP-treated cells showed obvious pyroptosis, which is the classic pyroptosis model [57, 58]. Here, PC12 cells were treated with LPS at different times and ATP for 1 hour to generate an inflammation-related pyroptosis model, which was utilized to assess the expression of HSF1 and NLRP3 in the classic pyroptosis model (Figure 5(a)). LPS treatment for 12 hours was chosen for further research. qPCR results revealed that the stimulation of LPS+ATP can induce more NLRP3 and IL-1*β* in PC12 cells transfected with *hsf1* siRNA than PC12 cells without *hsf1* siRNA interference (Figure 5(b)). Similar results were obtained in PC12 cells transfected with *hsf1* siRNA or plasmid. The mRNA expression of NLRP3 and IL-1*β* was inhibited by *hsf1* plasmid but enhanced by *hsf1* siRNA (Figure 6(a)). Western blot also proved that HSF1 could negatively regulate NLRP3 protein expression (Figures 6(b) and 6(d)). Similarly, *hsf1* siRNA enhanced the pyroptosis in PC12 cells, which could be reversed by *nlrp3* siRNA (Figures 6(c) and 6(e)).

### 3.5. HSF1 Regulates Pyroptosis Dependent on NLRP3 in PC12 Cells *In Vitro*

**Figure 7:**
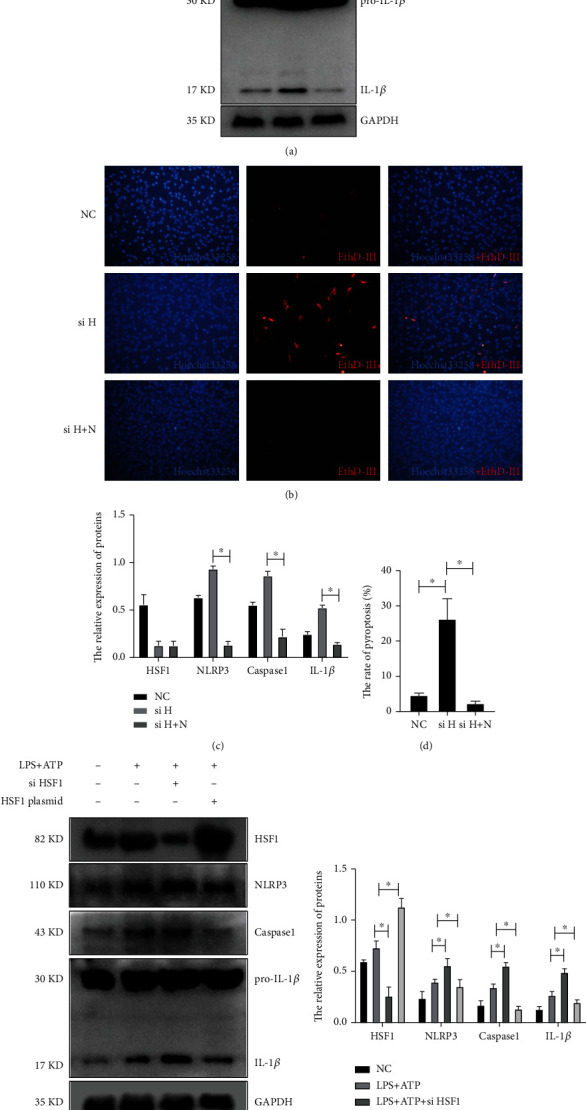
HSF1 can inhibit pyroptosis through NLRP3. (a, c) Western blot was used to detect the expression of the inflammatory cytokines and pyrolytic proteins in PC12 cells, ^∗^*p* < 0.05. (b, d) EthD-III staining (red) was labeled as pyroptosis, and Hoechst staining (blue) was tagged as nuclei. NC: negative control group; si H: *hsf1* silence group; si H+N: *hsf1* and *nlrp3* silence group. Experiments were repeated independently, ^∗^*p* < 0.05. (e, f) Western blot was used to detect the expression of the inflammatory cytokines and pyrolytic proteins in PC12 cells. NC: negative control group; LPS+ATP: LPS+ATP-treated group; LPS+ATP+si HSF1: LPS+ATP-treated PC12 cells transfected with HSF1 siRNA; LPS+ATP+HSF1 plasmid: LPS+ATP-treated PC12 cells transfected with HSF1 plasmid, ^∗^*p* < 0.05. Scale bar: 100 *μ*m, apply to all the panels.

The *nlrp3* siRNA was cotransfected into PC12 cells with *hsf1* siRNA. The real-time PCR and Western blot results showed that the upregulation of IL-1*β* and caspase1 in *hsf1* silence PC12 cells was reversed by transfecting with *nlrp3* siRNA (Figures 7(a) and 7(c)), and *hsf1* siRNA enhanced the pyroptosis in PC12 cells, which could be reversed by *nlrp3* siRNA (Figures 7(b) and 7(d)). This proved that HSF1 could inhibit the NLRP3-dependent pyroptosis. In the LPS+ATP-induced septic PC12 model, HSF1-transfected plasmid can inhibit the NLRP3 pathway, while HSF1 siRNA can enhance the expression of NLRP3 and IL-1*β* (Figures 7(e) and 7(f)).

## 4. Discussion

The major characteristics of sepsis are systemic inflammatory responses accompanied by multiple organ dysfunction syndromes. It has proved to cause more neutrophil infiltration in the lungs and kidneys of *hsf1*^−/−^ mice [56]. The *hsf1*^−/−^ septic mice exhibited a greater degree of lung, liver, and kidney tissue damage by increased fibrin:fibrinogen deposition compared with septic wild-type mice [52]. However, the degree of the impaired brain in *hsf1*^−/−^ septic mice was not certificated. The brain injury according to the acute phase of sepsis led to serious sequelae, which were the key factor of the prognosis of patients with sepsis. The brain could be impaired by increasing inflammatory cytokines in sepsis. Unfortunately, there is no effective way to prevent or even treat this grievous disease, and the pathogenesis of brain injury in sepsis remains to be clarified [59, 60]. The occurrence of brain injury in sepsis is closely related to inflammation, and inhibiting the inflammatory factors' expression in brain tissues could alleviate sepsis encephalopathy [13]. Sepsis induces significant brain disorders and the dysfunction of neurons and synaptic plasticity of the cerebral cortex and hippocampus [61, 62]. The hippocampus and cortex are vulnerable to cell death; therefore, it is necessary to prevent the hippocampus from cell death in septic patients [61].

Cell death is controlled partially by RCD pathways, which comprise apoptosis, necroptosis, ferroptosis, pyroptosis, etc. [53, 63–72]. Pyroptosis has been proved to be closely related to sepsis-induced organ damage, but whether inhibiting pyroptosis could be a possible therapy tactic for the brain in sepsis is starved for more evidence [73, 74]. In this study, we focus on exploring the candidate molecules or pathways that could regulate pyroptosis. The NLRP3-dependent caspase1/IL-1*β* pathway is the key regulator of pyroptosis [75], and the upregulation of NLRP3 expression can promote inflammation and pyroptosis [38]. While pyroptosis is the inflammatory form of RCD, it can release IL-1*β* and IL-18 in the early stages to initiate the event of sepsis [76]. HSF1, the key regulator of heat shock response, prevents harmful stimulation, which can inhibit the expression of NLRP3 [77, 78]. HSF1 can protect the lung, liver, and kidney from multiorgan dysfunction syndrome induced by sepsis, but the function and expression in the brain had not been demonstrated [52, 56]. As a result, the relationship between HSF1 and NLRP3 in the CLP septic mouse model was investigated. Unexpectedly, we found that HSF1 is intrinsically increased in the hippocampus of CLP septic mice (Figure 1) and positively related to the changes of inflammatory factors NLRP3, caspase1, and cleaved IL-1*β*, which is significantly different from some previous reports claiming HSF1 could downregulate NLRP3 [50, 77, 79].

Although the expression of HSF1 increased in the septic mouse model, we are still assured that the elevated HSF1 antagonizes the sepsis in the CLP model. To certify the exact roles of HSF1 in the brain of sepsis, wild-type mice and *hsf1*^−/−^ mice were used to build the CLP model. The brain tissue collected from differently treated mice was used for functional exploration. The EthD-III experiments showed that HSF1 was also negatively related to pyroptosis. The pyroptosis in brain tissue cells significantly increased in the *hsf1*^−/−^ CLP model compared with the *hsf1*^+/+^ CLP model, while the *hsf1*^−/−^ mice also showed more pyroptosis in cells of brain tissue than *hsf1*^+/+^ mice (Figure 2). In further research, PC12 cells were utilized to simulate the neuron model, which revealed that *hsf1* siRNA could upregulate pyroptosis while HSF1 plasmid can inhibit the pyroptosis (Figure 6). It is confirmed that pyroptosis could initiate sepsis [38, 76, 80], implying that HSF1 could alleviate sepsis-induced brain dysfunction by blocking the initiation event of sepsis by inhibiting pyroptosis.

Interestingly, the level of NLRP3/caspase1/cleaved IL-1*β* was significantly elevated in the *hsf1*^−/−^ CLP mice compared with *hsf1*^+/+^ CLP mice (Figures 3 and 4). These results explained that the elevated expression of HSF1 in the CLP model could protect mice from inflammation factors (Figures 1, 3, and 4), and the lack of HSF1 makes the inflammation aggressive in the CLP mice (Figures 3 and 4). The same results are also found in the *in vitro* model, and *nlrp3* mRNA increased in the PC12 cells transfected with *hsf1* siRNA (Figure 5). Further research revealed that the *nlrp3* siRNA can reverse *hsf1* siRNA that enhanced the pyroptosis in PC12 cells (Figure 7). Collected all together, these data suggested that HSF1 might be a protective factor of pyroptosis by inhibiting NLRP3. The mechanism of HSF1-regulated NLRP3 was reported in a few articles. HSF1 could indirectly control the expression of NLRP3 by activating Snail and regulating the TRX1/TXNIP and TRX1/ASK1 complexes or promoting *β*-catenin translocation from the cytoplasm to the nucleus, which inhibits XBP1 activation in response to TLR/TRAF6 stimulation in macrophages by enhancing *β*-catenin transcriptional activity [50, 77]. Our recent research proved that HSF1 was involved in the activation of the NLRP3 inflammasome in septic acute lung injury (ALI) [81]. We proved that HSF1 could suppress NLRP3 inflammasome activation in transcriptional and posttranslational modification levels and that HSF1 can inhibit caspase1 activation and IL-1*β* maturation *via* inhibiting the NLRP3 pathway [81]. This work supports previous results and indicates that HSF1 prevents brain injury from sepsis by inhibiting the sepsis-induced pyroptosis through the NLRP3-dependent caspase1/IL-1*β* pathway.

## 5. Conclusion

Our works showed that HSF1 plays an important role in sepsis-induced brain injury by regulating NLRP3. Although the exact mechanism of HSF1 inhibiting the NLRP3/caspase1/cleaved IL-1*β* pathway was not considered in this study, we conclude that HSF1 prevents brain injury from sepsis by inhibiting the sepsis-induced pyroptosis through the NLRP3-dependent caspase1/IL-1*β* pathway, suggesting HSF1 as a potential molecular target for treating brain injury in sepsis clinical studies.

## Figures and Tables

**Table 1:** The primer sequences used for qPCR.

Primer	Sequence
*Mouse hsf1* forward:	5′-GGACATAAAAATACGCCAGGAC-3′
*Mouse hsf1* reverse:	5′-GAGCTTGTTGACAACTTTTTGC-3′
*Mouse il*-*1β* forward:	5′-CACTACAGGCTCCGAGATGAACAAC-3′
*Mouse il*-*1β* reverse:	5′-TGTCGTTGCTTGGTTCTCCTTGTAC-3′
*Mouse nlrp3* forward:	5′-ATTACCCGCCCGAGAAAGG-3′
*Mouse nlrp3* reverse:	5′-TCGCAGCAAAGATCCACACAG-3′
*Mouse β*-*actin* forward:	5′-TCACTATTGGCAACGAGCGGTTC-3′
*Mouse β*-*actin* reverse:	5′-CAGCACTGTGTTGGCATAGAGGTC-3′
*Rat hsf1* forward:	5′-TCTGAGGGAGATGACTACACGGATG-3′
*Rat hsf1* reverse:	5′-AAGCCAGCCTGACAACTGAAACC-3′
*Rat il*-*1β* forward:	5′-AATCTCACAGCAGCATCTCGACAAG-3′
*Rat il*-*1β* reverse:	5′-TCCACGGGCAAGACATAGGTAGC-3′
*Rat nlrp3* forward:	5′-GAGCTGGACCTCAGTGACAATGC-3′
*Rat nlrp3* reverse:	5′-AGAACCAATGCGAGATCCTGACAAC-3′
*Rat β*-*actin* forward:	5′-GGTCAGGTCATCACTATCGGCAATG-3′
*Rat β*-*actin* reverse:	5′-CAGCACTGTGTTGGCATAGAGGTC-3′

## Data Availability

The data used to support the findings in this study are available from the corresponding author upon reasonable request.

## Abbreviations

ATP: Adenosine triphosphate
BBB: Blood-brain barrier
CLP: Cecal ligation and puncture
CNS: Central nervous system
DMEM: Dulbecco's modified Eagle's medium
HSPs: Heat shock proteins
HSR: Heat shock response
IL-1*β*: Interleukin-1*β*
LPS: Lipopolysaccharides
NLRP3: NLR family pyrin domain-containing 3
PBS: Phosphate-buffered saline
PCR: Polymerase chain reaction
ROS: Reactive oxygen species
RT: Room temperature
SAE: Sepsis-associated encephalopathy
SDS-PAGE: Sodium dodecyl sulfate-polyacrylamide gel electrophoresis
siRNA: Small interfering RNA.
